# Parallelization of Three Dimensional Cardiac Simulation on GPU

**DOI:** 10.3390/biomedicines12092126

**Published:** 2024-09-19

**Authors:** Qin Li, Xin Zhu, Wenxi Chen

**Affiliations:** 1 Graduate School of Computer Science and Engineering, The University of Aizu, Aizu-Wakamatsu 965-8580, Fukushima, Japan; d8212105@u-aizu.ac.jp; 2 Department of AI Technology Development, M&D Data Science Center, Tokyo Medical and Dental University, Chiyoda 101-0062, Tokyo, Japan

**Keywords:** simulation, modeling, cardiac electrophysiology, parallelization, GPU

## Abstract

Background: The simulation of electrophysiological cardiac models plays an important role in facilitating the investigation of cardiac behavior under various conditions. However, these simulations often require a lot of computational resources. Methods: To address this challenge, this study introduced a method for speeding up three-dimensional cardiac simulations using GPU parallelization. A series of optimizations was introduced, encompassing various aspects such as data storage, algorithmic enhancements, and data transfer. Results: The experimental results reveal that the optimized GPU parallel simulations achieve an approximate 50-fold acceleration compared with their CPU serial program. Conclusion: This investigation substantiates the considerable potential of GPUs in advancing the field of cardiac electrophysiology simulations.

## 1. Introduction

In recent years, rapid advances in computational capabilities and improvements in modeling have highlighted the significance of simulations in physiology. Compared with traditional physiological experiments, cardiac simulations provide a platform for researchers to investigate heart behavior under various controlled conditions comprehensively. This computational approach allows to study complex heart dynamics that might be hard or impossible to achieve through traditional laboratory experiments.

The simulation of a cardiac model can be thought of as a reaction–diffusion system. The reaction part explains how action potentials (APs) originate and change within individual heart muscle cells. In contrast, the diffusion part represents how these APs spread across a spatial area defined by a two-dimensional (2D) or three-dimensional (3D) mesh in nearby regions.

In cardiac simulations, the reaction phase is usually characterized by ordinary differential equations (ODEs) showing ion channels’ complex interactions influencing action potential (AP) dynamics within myocardial cells. On the other hand, the diffusion phase is represented by partial differential equations (PDEs) that describe how APs propagate from one cell to neighboring cells. The interaction between the reaction and diffusion phases occurs at discrete time steps, encapsulating the essential cardiac simulation process.

The complexity of reaction–diffusion systems requires numerical methods to solve them. Direct algorithms for determining the solutions of most ODEs and PDEs of this nature are still lacking [1]. As a result, simulations require iterative algorithms such as the Euler method [2] or the Runge–Kutta method [3]. However, these methods require very small time steps, leading to significant computational overhead.

The computational demands involved in simulating the heart are substantial, primarily due to the complex geometries of cardiac tissues. Three-dimensional cardiac models can encompass tens of millions to billions of discrete elements, making the simulation of their intricate dynamics a formidable computational task [4]. Consequently, the computational resources and processing power required for these simulations have significantly increased, necessitating the adoption of advanced computational paradigms.

Researchers often employ parallelization techniques to utilize available computational resources to accelerate simulations fully. These parallel methods generally fall into two categories. Initially, parallelization based on High-Performance Computing (HPC) dominated the field. For instance, Zhu et al. [5] constructed a whole-heart model using a four-node cluster of shared-memory computers. Similarly, Niederer et al. [6] and Pavarino et al. [7] utilized HPC algorithms to simulate human cardiac electrophysiology. While these approaches showed significant performance improvements, the increasing costs associated with HPC infrastructure and maintenance have limited their widespread adoption. With the development of powerful parallel computing devices such as Many Integrated Cores (MIC), Graphics Processing Units (GPUs), and Field-Programmable Gate Arrays (FPGAs), the focus of parallelization has shifted from HPC to these platforms. Various techniques have been proposed for utilizing these devices. Adon et al. [8] and Othman et al. [9] implemented cardiac simulations on FPGA platforms, while Yang et al. [10] employed MIC to address cardiac models. As GPUs have evolved, the emphasis in parallel computing [11,12,13,14] has gradually shifted towards GPU platforms. The early stages of GPU parallelization are constrained by hardware limitations, such as limited computational power and video memory, which restrict the scale of simulations. Consequently, early GPU parallelism often required the collaborative use of multiple GPU cards to execute simulation tasks efficiently. Research in this area primarily focused on optimizing task scheduling and workload distribution to achieve efficient computation [15,16].

Today, advancements in hardware technology have made it feasible to conduct extensive simulations on GPUs within personal computers. However, due to architectural differences between CPUs and GPUs, directly porting CPU-based cardiac model programs to GPUs is not straightforward. To address this, Lionetti et al. [17] and Amorim et al. [18] designed platforms for automatically transferring cardiac simulation code from CPUs to GPUs. Nevertheless, these auto-generated GPU codes often need more optimization and have limited efficiency. Qiu et al. [1] optimized cardiac electrophysiology simulations on a 2D mesh using GPUs by replacing individual node ODE calculations with a pointer movement method, obtaining action potential values solely through table look-up. While this approach yielded remarkable acceleration, it overlooked the impact of voltage gradients on cell parameters. Liu et al. [4] utilized Nvidia’s embedded devices and used the shared memory between RAM and GPU memory to achieve acceleration on CPU–GPU platforms. Although highly efficient, this approach can lead to frequent memory swaps in personal computers due to the separation of RAM and GPU memory, severely limiting performance.

In addition to the above, many recent studies have focused on accelerating cardiac simulations. For instance, Couto et al. [19] proposed a second-order numerical solving method specifically for cardiac simulation and implemented it using OpenMP for multithreading, achieving an acceleration ratio of approximately 4.6 on a 6-core processor. Sakka et al. [20] compared three methods for accelerating cardiac simulations: multithreading, vectorization, and GPU computing. The experimental results showed that the parallel efficiency of GPU computing and vectorization is three times that of multithreading. This result demonstrates that using GPU for cardiac acceleration is a reasonable and efficient choice.

Regarding the use of GPUs, some of this research concentrates on parallelizing simulations on GPU clusters, emphasizing task scheduling and allocation. For instance, Viola et al. [21] used CUDA Fortran to port their multi-physics solver to the GPU architecture. Based on this, they completed the computation of fluid-structure–electrophysiology interaction on a heterogeneous platform, utilizing both CPU and GPU for acceleration. Chen et al. [22] provided a coupling physics model and simplified the system equations. Additionally, they parallelized their method on the GPU. However, most of the study focused on their physical system, and the GPU parallelization aspect was not explored in depth. Due to differences in the specific implementation of the PDE component, other studies have limited relevance to our work. Given the high cost of GPU clusters, our goal is to achieve parallelization of our three-dimensional simulation algorithm on a single GPU.

This study introduced a method for simulating three-dimensional computational cardiac models within a GPU framework. We have implemented various parallelization strategies to optimize the simulation program, focusing on improving runtime efficiency and memory utilization. Our investigation shows that GPU parallelization is highly advantageous for large-scale cardiac simulations, significantly reducing runtime and achieving impressive acceleration compared with CPU-based simulations. The integration of GPU computing dramatically speeds up the simulation process. It enhances computational throughput, making it an attractive option for researchers looking to improve the efficiency of cardiac modeling and analysis.

### 1.1. Model

An action potential is characterized by a rapid rise and subsequent fall in voltage, or membrane potential, across a cellular membrane, following a distinct pattern [23]. A sufficient current is necessary to initiate a voltage response in a cell membrane; if the current is inadequate to depolarize the membrane to the threshold level, an action potential will not be generated.

Pacemaker cells in the sinoatrial (SA) node intrinsically and rhythmically initiate action potentials within the heart. These action potentials are then propagated throughout the heart by myocardiocytes, which are cardiac muscle cells that contract while conducting the current to neighboring cells [24].

Based on Hodgkin and Huxley’s theory [25] concerning single-cell models, the structure of a cell model can be abstracted into a circuit diagram. The governing equation of this circuit can be expressed as follows:

(1) $$I=C_{M}\frac{dV}{dt}+I_{\mathrm{ion}}$$

where *I* signifies the total current, $C_{M}$ denotes the cell membrane capacity, *V* represents the voltage, and $I_{\mathrm{ion}}$ corresponds to the ion current.

The total current *I* is conventionally set to zero for a single cell. However, to induce an action potential initiation in a cell, a stimulation current denoted as $I_{\mathrm{stim}}$ is introduced to Equation (1). This adaptation transforms the equation as follows:

(2) $$\frac{dV}{dt}=-\frac{1}{C_{M}}\left(I_{\mathrm{ion}}+I_{\mathrm{stim}}\right)$$

In various cell models, the ion current has a unique mathematical representation. Generally, the essence of an individual ion current can be captured by the following equation:

(3) $$\frac{du}{dt}=f\left(u,V\right)$$

Here, *u* denotes the parameters of a specific ion channel, and the function *f* depicts the relationship between the voltage *V* and the parameters *u*.

By integrating these two equations, a comprehensive description of a cell model emerges, providing a precise representation of its behavior.

### 1.2. Three-Dimensional Model

The bidomain model is well-known for its high precision in representing cardiac activity but comes with significant computational complexity [26]. This research aims to avoid these complex issues by using the monodomain model to conduct three-dimensional cardiac simulations. The following equation defines the monodomain model:

(4) $$\frac{dV}{dt}=\nabla \cdot \left(D\nabla V\right)-\frac{I_{\mathrm{ion}}}{C_{m}}$$

Here, *V* signifies the membrane voltage, *t* symbolizes time, ∇ designates the gradient operator, *D* represents the diffusion tensor, $I_{\mathrm{ion}}$ aggregates all transmembrane ion currents, and $C_{m}$ corresponds to the transmembrane capacitance. This equation integrates ODEs and a PDE, forming a complex nonlinear system.

To solve this equation, we employ the operator splitting technique [27], which involves separating the equation into two distinct parts:

(5) $$\frac{dV}{dt}=\nabla \cdot \left(D\nabla V\right)$$

(6) $$\frac{dV}{dt}=-\frac{I_{\mathrm{ion}}}{C_{m}}$$

Equation (5) involves a partial differential equation (PDE) that describes the propagation process. Equation (6) represents a single-cell model characterized by ordinary differential equations (ODEs), depicting intracellular electrical activity.

For the reaction component, we utilize the Luo Rudy 1991 model [28] as our chosen cell model due to its detailed mathematical representation of the ventricular cardiac action potential. This model is preferred for its comprehensive depiction of cellular electrical phenomena.

For the diffusion component, traditional finite difference methods are insufficient for resolving PDEs due to the varying inter-node distances within our 3D mesh. Therefore, we employ a lumped mass method to compute the distance ratio based on the spatial coordinates of the nodes and their adjacent neighbors.

### 1.3. GPU Programming

A Graphics Processing Unit (GPU) is a specialized electronic circuit designed to rapidly manipulate and modify memory, facilitating the accelerated generation of images within a frame buffer intended for display on output devices [29]. Due to their inherently parallel architecture, contemporary GPUs significantly outperform general-purpose Central Processing Units (CPUs) for algorithms that require parallel processing of substantial data blocks.

The Compute Unified Device Architecture (CUDA), introduced and developed by NVIDIA, is a parallel computing platform and Application Programming Interface (API) model. CUDA enables the utilization of GPUs as co-processors to implement parallel computations. Specifically, code executed directly on the GPU chip is defined as a kernel function. However, prior to the execution of a CUDA kernel, users must manually allocate and initialize memory space within the Video RAM (VRAM). Following the completion of a kernel function, the resultant data requires manual transfer from VRAM to the system RAM. The structural framework of a typical CUDA program follows these steps:

1. Allocate memory space on VRAM.
2. Copy data from RAM to VRAM.
3. Executing kernel functions.
4. Copy data from VRAM to RAM.
5. Free space allocated in Step 1.

The GPU board contains different types of storage media in the VRAM, such as registers, shared memory, and global memory. Registers provide the fastest access but have limited availability and are mainly allocated to local variables within CUDA kernels. Shared memory, although slightly slower than registers, allows communication among threads within the same block. On the other hand, global memory, while slower, has a much larger capacity and is accessible by all GPU threads.

In a CUDA program, the fundamental execution unit is the thread, and these threads are organized into blocks. Threads within a block can communicate through shared memory. When a CUDA program runs on a GPU, blocks with multiple threads are assigned to Streaming Multiprocessors (SMs) and operate in Single Instruction, Multiple Data (SIMD) mode. Each thread executes the same instruction, guided by the shared Instruction Unit, while processing its individual data stream from device memory to on-chip cache memories and registers. It is important to note that blocks and threads are software abstractions. The block and thread size must be manually specified in a GPU program. Since the optimal values vary depending on the application, there is no fixed setting. Before the full simulation, we experiment with combinations and conduct short parallel simulations to determine the appropriate block and thread settings.

In the event of a cache miss within a running block, the context switches immediately to the following block to minimize the impact of the cache miss. Therefore, the strategic arrangement of the number of blocks per grid and the number of threads per block in a CUDA program significantly influences overall program efficiency.

## 2. Methods

### 2.1. Three-Dimensional Mesh

**Figure 1:**
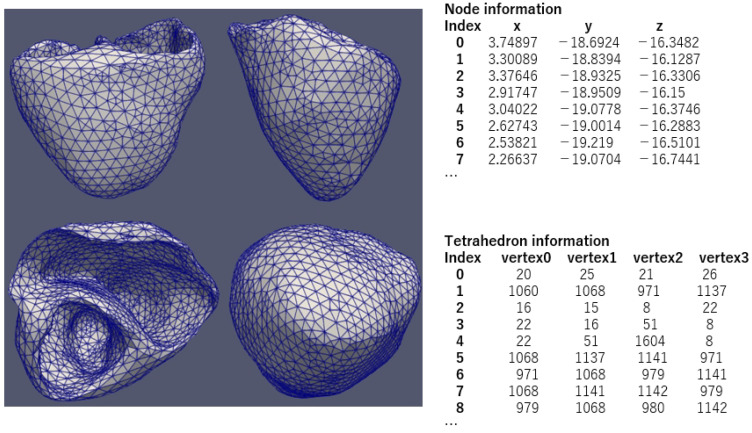
The structure of a simulation mesh.

This study used a three-dimensional (3D) mesh to intricately model the cardiac system. The mesh comprises tetrahedrons, and its architecture’s graphical representation can be seen in Figure 1. The mesh comprises a total of 4168 nodes and 17,939 tetrahedrons. Each node is defined by a set of coordinates {x, y, z}, which indicate its position in a 3D Cartesian coordinate system. The tetrahedrons are defined by four distinct node indices representing their vertices. This detailed representation of the 3D mesh effectively captures the intricate details of the physiological system being studied.

### 2.2. Preprocessing

#### 2.2.1. Mesh Storage Improvement

**Figure 2:**
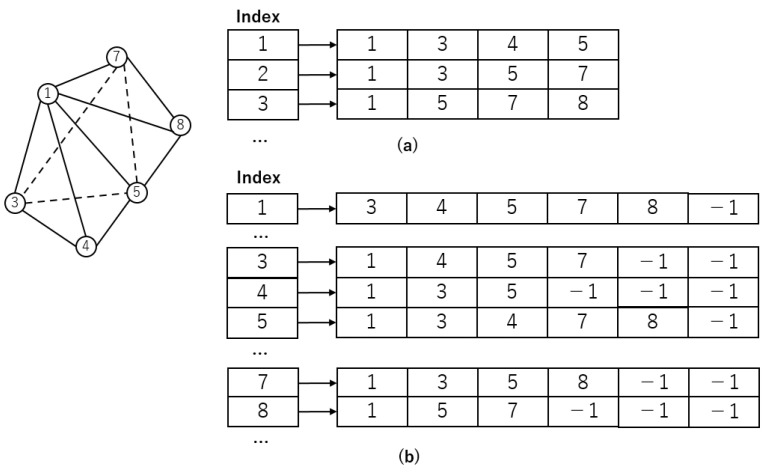
The framework of mesh storage. (**a**): Mesh storage without adjacency list. (**b**): Mesh storage using adjacency list.

In our investigation, the 3D mesh we use consists of tetrahedrons, each defined by four vertices. Conventionally, a data array stores this mesh information, as shown in Figure 2a. In this array structure, each element holds the indices of the four vertices that comprise a tetrahedron. While this method effectively represents the geometric arrangement of the 3D grid, it encounters challenges during the simulation’s diffusion and propagation phases. Specifically, every node needs access to the action potential of its neighboring nodes.

If we adopt the above storage strategy, accessing the neighboring nodes of a specific node requires traversing through all the tetrahedra. The process involves checking whether the current node belongs to the tetrahedron’s vertices for each tetrahedron. If it does, the remaining three nodes are considered the neighbors of that node. Although this method has the advantage of obtaining three neighboring nodes at once, it requires an additional array for each node to keep track of its visits, preventing redundant computations of the same node. This not only slows down the execution speed but also adds complexity to the programming.

Using this method, the time complexity of finding neighboring nodes is O(n), where n represents the number of tetrahedra in the mesh. As the simulation scale increases, the process of finding neighboring nodes becomes progressively slower, highlighting the inefficiency of this approach for large-scale simulations.

In graph theory, graph information is typically stored using an adjacency list or an adjacency matrix [30]. This study introduces an enhanced adjacency list to store mesh information, as adjacency matrices require substantial storage space. Traditional adjacency lists consist of arrays and linked lists. However, these linked lists could disrupt data caching and prefetching techniques when executing GPU-parallelized programs in a Single Instruction, Multiple Data (SIMD) fashion. To address this, fixed-length arrays instead of traditional linked lists offer significant runtime performance and implementation advantages.

The algorithm for mesh storage using the enhanced adjacency list is shown in Algorithm 1. For each tetrahedron in the mesh, a nested loop iterates through the four vertices of the tetrahedron. For each vertex pair, node u is added to the adjacency list of node v, and vice versa. Additionally, the counts of neighboring nodes for both nodes are updated. This approach transforms the storage of interconnections between nodes in the mesh. Using an adjacency list results in additional memory usage compared with the original storage method, as shown in Figure 2b. However, it allows each node to quickly access its neighboring nodes, enhancing the efficiency of the simulation process.

The improved storage method already stores the node IDs within each node’s adjacency list. This means accessing neighboring nodes is conducted by going through that node’s adjacency list. This approach has a time complexity of O(m), where m is the number of neighboring nodes for that specific node. As a result, this method significantly speeds up the process of accessing neighboring nodes, thereby improving simulation efficiency.

Additionally, as shown in Algorithm 1, constructing the adjacency list requires traversing the tetrahedra once. Therefore, enhancing this storage method does not add any extra runtime overhead. This efficiency gain and the ease of accessing neighboring nodes make the improved storage method beneficial for large-scale simulations.
**Algorithm 1:** Mesh storage
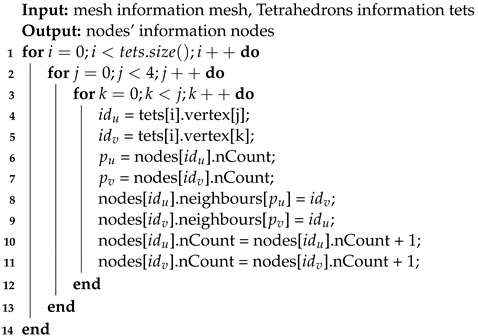


#### 2.2.2. Node Index Regeneration

In order to make the most of Single Instruction, Multiple Data (SIMD) processing, GPUs use a multi-level cache strategy in their architecture. Accessing the cache is much faster than accessing global memory, often by several orders of magnitude. Therefore, optimizing the cache and increasing cache hit rates can significantly improve overall runtime performance. To achieve it, it is essential to reorder node indices strategically.

Certain tetrahedra in the mesh often have significantly different numberings of the four vertices. It means that GPU data access may heavily depend on global memory access. The mesh is preprocessed before the simulation to reduce this dependence and minimize its impact.

During preprocessing, the nodes are renumbered using the breadth-first search (BFS) method, an essential technique described in the provided pseudocode as shown in Algorithm 2. The BFS method uses a queue to access nodes based on their proximity to a given node, following a first-in, first-out (FIFO) data structure. This ensures that elements are dequeued in the same order they were enqueued, starting with an initial node as the starting point.
**Algorithm 2:** Node Id Generation
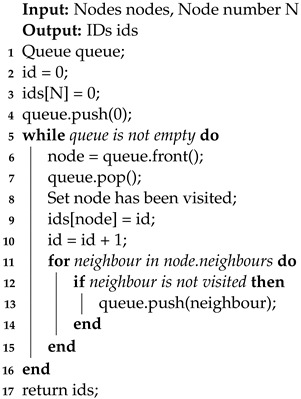


**Figure 3:**
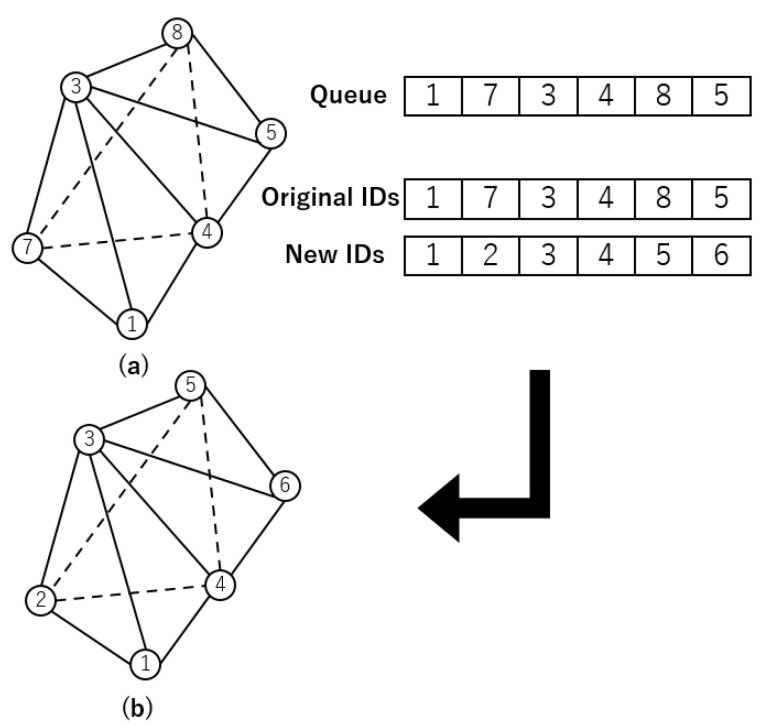
The example diagram of node index regeneration. (**a**): Node index organization before node index regeneration. (**b**): Node index organization after node index regeneration.

Figure 3b shows this optimized arrangement of node indices. This reordering significantly increases the likelihood of successful cache entries, improving computational efficiency. Additionally, since each node is enqueued and dequeued only once, the algorithm’s time complexity converges asymptotically to O(n). This means that even in large-scale simulations, the execution of this process does not incur substantial time overhead.

### 2.3. CPU Implementation

The framework for three-dimensional cardiac electrophysiology simulations running on a CPU is shown in Algorithm 3. Before starting the simulation, several preprocessing steps are performed. These steps include loading the mesh, calculating distance coefficients, and configuring relevant simulation parameters. Then, the numerical solving process for iterative updates takes place.
**Algorithm 3:** CPU Simulation
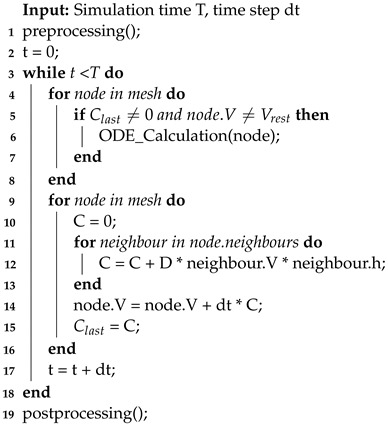


Throughout the iterations, the simulation process adapts to the chosen numerical solving method, such as the widely used Forward Euler method. This method, exemplified here, guides the program through each cell node in the mesh within each time step. The process begins with the calculation of ordinary differential equations (ODEs) for each cell node, involving the updating and iterating of the cell’s parameters for the current time step using the parameters from the previous time step, reflecting the cell’s inherent electrophysiological activity.

ODE calculations make up a significant portion of the computational load in this three-dimensional simulation implementation. To optimize this, a small but effective strategy is employed. The ODE equations show that when no external stimulating current exists, the cells are resting, during which none of the cell parameters change. Thus, when the stimulating current is zero, ODE calculations can be skipped. To implement this in the program, a parameter keeps track of the sum of membrane potential differences from neighboring nodes in the previous time step. This parameter is evaluated when performing ODE calculations in the current time step. If it is very close to zero and the membrane potential at the current node is at the resting potential, the current node is considered resting, allowing the ODE calculations to be skipped.

The program follows a systematic approach to updating the potential values of neighboring nodes. It first identifies all neighboring nodes for each cell node and then updates their potential values based on the specific numerical iteration method. For instance, in the case of the Euler method, the potential value of the neighboring node is multiplied by the distance coefficient between that node and the current node and the propagation coefficient. These products are then added together and collectively updated onto the potential value of the current cell node, thereby propagating the potential values.

### 2.4. GPU Implementation

When employing GPUs for parallel acceleration, a prevalent strategy is task partitioning. This involves assigning a portion of the task to each core on the GPU to leverage its numerous cores. However, in the context of cardiac simulations, the iterative solving of differential equations introduces a temporal dependency between data from consecutive time steps, rendering temporal task partitioning impractical. On the contrary, in the spatial domain, the electrophysiological activity of each cell node only involves the cell’s parameters. Hence, spatial task partitioning, where each core of the GPU is responsible for computing a portion of the cell nodes, emerges as a more effective and efficient parallelization approach.

The algorithmic framework is shown in Algorithm 4. The preprocessing phase of GPU parallel cardiac simulations is similar to CPU-based simulations. However, the main difference is allocating the appropriate video memory on the GPU during the preprocessing stage, as the simulation program runs on the GPU. Additionally, relevant simulation parameters must be transferred from CPU to GPU video memory.
**Algorithm 4:** GPU parallelization
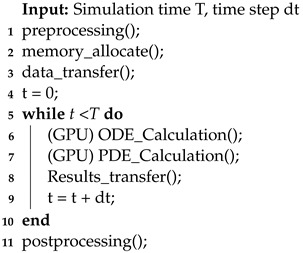


In CPU simulations, ODEs and PDEs are calculated by iterating through each cell node in the entire mesh. However, in GPU parallel simulations, this process is replaced by GPU kernel functions. Typically, the program executes two kernel functions within each time step: one for calculating a cell’s intrinsic electrophysiological activity and another for calculating potential propagation. The calculation methods are generally similar to CPU programs and are determined by the corresponding numerical solving methods.

After both computational processes are finished, the results should promptly be returned to the CPU. Then, the simulation calculates the next time steps, repeating this process until the total simulation time meets the predetermined requirements. Algorithm 5 and 6 illustrate the principle behind GPU kernel functions. Despite the GPU having a significant increase in the number of cores compared with the CPU, it is uncertain whether each node is allocated a distinct core for computation due to the scale of the simulation. Therefore, specific cores are responsible for managing concurrent computations for multiple nodes.
**Algorithm 5:** ODE_Calculation
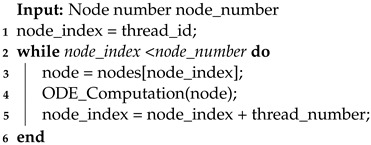


**Algorithm 6:** PDE_Calculation

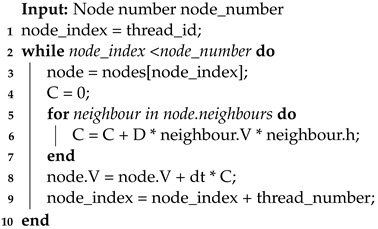



Consider a thread represented by the ID x. This thread performs computations for nodes with IDs x + kN, where N denotes the number of threads, and k spans non-negative integers (k = 0, 1, 2, 3, …). This method is designed to prevent address conflicts. Adhering to this addressing scheme ensures that each thread is dedicated to the computation of distinct nodes, fostering the active participation of all threads in the computation process. Furthermore, this approach guarantees that the differences in the number of nodes computed by each thread are maintained at one, thereby maintaining balance among all threads and promoting computation speed and efficiency improvements.

### 2.5. Improved Data Storage

**Figure 4:**
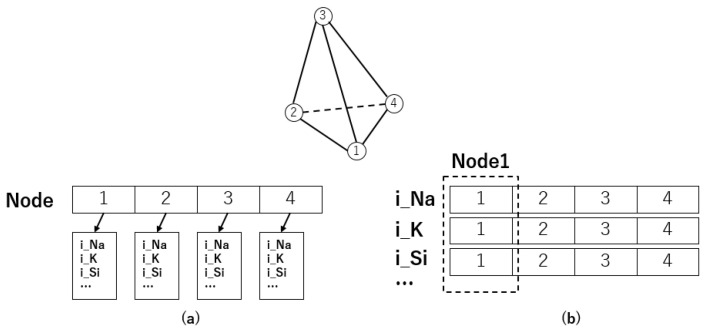
Data structure of cell model. (**a**): Data arrangement using structure/class. (**b**): Data arrange using array.

In traditional CPU serial programs, improving code readability and enabling code reuse often involves using a structured approach with classes or structures to encapsulate a model containing all related parameters. However, transitioning this methodology into GPU parallelism can result in significant resource inefficiencies. In a GPU thread warp, all threads execute a unified instruction simultaneously. When the GPU accesses global memory, it retrieves data in 32-byte blocks. In the storage method depicted in Figure 4a, when data are retrieved, the GPU loads subsequent parameters into the cache, which hinders the execution of instructions for other threads. This causes a decrease in cache hit rates and frequent global memory accesses, thus reducing the overall efficiency of the simulation.

To solve this problem, we use a data storage design, as depicted in Figure 2b, during GPU parallelism. In this design, each parameter in the model is stored separately. This allows the GPU to gather the same parameters from neighboring nodes and put them into the cache during global memory reads. As a result, this reduces access conflicts by other threads within the thread warp. Furthermore, we identify and allocate constant parameters in texture memory to optimize GPU memory usage.

**Figure 5:**
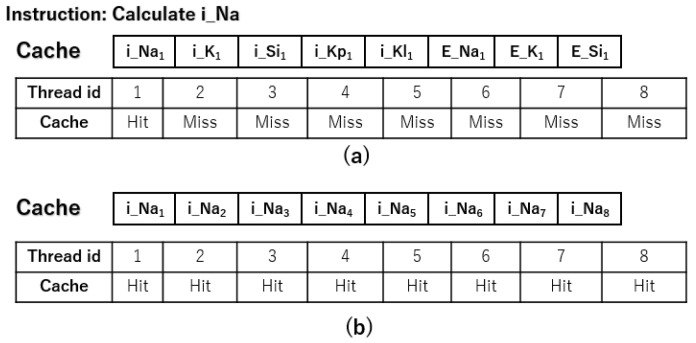
The diagram of cache usage. (**a**): Cache hit rate without improved data storage. (**b**): Cache hit rate with improved data storage.

For example, let’s say the current thread needs to calculate $i_{\mathrm{Na}}$. In SIMD mode, all threads within a warp will execute this instruction. When thread 1 performs the calculation and finds that the variable $i_{\mathrm{Na}}$ is not in the cache, it results in a cache miss. As a result, the GPU fetches $i_{\mathrm{Na}}$ from global memory into the cache. Following prefetching techniques, the GPU will bring in a portion of data after $i_{\mathrm{Na}}$ into the cache for future access. If the data are stored as shown in Figure 4a, the GPU will load other parameters of cell node 1 into the cache. However, the other threads do not need this part of the data for their $i_{\mathrm{Na}}$ calculations but require the $i_{\mathrm{Na}}$ value for their respective nodes. This causes cache misses for the other threads, leading to repeated global memory accesses, as illustrated in Figure 5a.

On the other hand, if we use the storage method depicted in Figure 2b, when we read the value of $i_{\mathrm{Na}}$ for node 1, it also brings in the $i_{\mathrm{Na}}$ values of other nodes into the cache. As shown in Algorithm 5, in this case, other threads can directly access the data from the cache, which reduces the need for frequent global memory accesses. This optimization improves overall simulation efficiency by increasing cache hit rates and minimizing memory access latency.

### 2.6. Improved Algorithm

Computing the ordinary differential equation (ODE) and partial differential equation (PDE) components separately in the numerical solution process of reaction–diffusion equations is essential. The PDE computation depends on the newly derived membrane potential values from the ODE calculations. This means that the execution of PDE calculations at each time step relies on completing ODE calculations for all nodes.

For first-order numerical methods such as the Forward Euler method, each time step involves three kernel functions. The first function calculates the ODE, the second calculates the PDE, and the third combines and updates the results of the first two functions. These functions are executed in sequence. However, each time step requires nine kernel functions for high-order numerical methods like the Runge–Kutta method. The first eight functions perform calculations for the first to fourth order, while the last function oversees the merging. This approach increases GPU resource overhead, and the creation and termination of many functions lead to longer runtime. Moreover, frequent switching between ODE and PDE solutions can decrease cache hit rates.

To enhance performance, this study proposes modifying the PDE solution approach. Specifically, instead of using the current time step’s ODE calculation outcome, data from the previous time step’s ODE computation is utilized during the PDE calculation. This means that the impact of the PDE on action potentials is postponed by a duration of Δt. By adopting this strategy, the ODE and PDE solutions are fully separated, eliminating any data interdependencies. For example, when using the Forward Euler method during the potential propagation phase, integrating the ODE and PDE calculations into a single kernel function is possible using potential data from neighboring nodes from the previous time step, as shown in Algorithm 7. When accessing the potential data of neighboring nodes, the value $V_{\mathrm{last}}$ is used for computation. The benefit of this approach is that when calculating potential propagation at the current node, there is no need to wait for the ODE portion of its neighboring nodes to finish computing, resulting in an acceleration effect. Although the improved algorithm still requires a synchronization process to update the potential from the previous step, this update is executed only once at each step. In contrast, using the unimproved approach with higher-order numerical methods would require multiple kernel function executions or synchronizations within a kernel function at each time step to ensure data consistency.
**Algorithm 7:** AP_Calculation
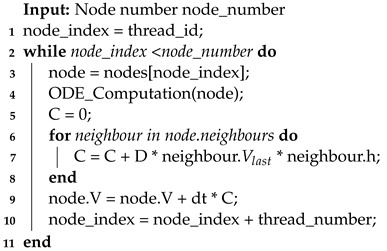


### 2.7. Async Data Transfer

When running simulations on a Graphics Processing Unit (GPU) instead of a Central Processing Unit (CPU), the data processed on the GPU must be sent back to the CPU before further processing and analysis can occur. In numerical algorithms that involve multiple iterations, such as those used for solving ODEs and PDEs, failure to promptly transfer data to the CPU can result in overwriting by results from subsequent time steps. An alternative approach is to store data in the GPU’s local memory and transfer it as a unified entity at the end of the simulation. However, this method leads to a linear increase in GPU memory requirements relative to simulation time. This impacts the simulation’s scale and highlights the importance of efficient data storage and transmission.

**Figure 6:**
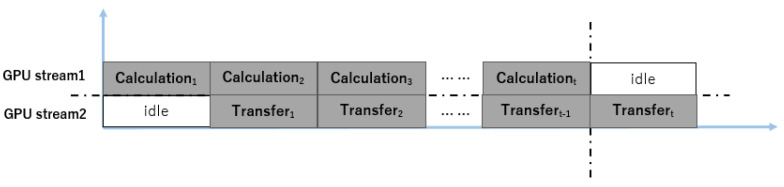
The framework of asynchronous data transfer.

As shown in Table 1, the experimental results suggest that data transmission significantly contributes to the overall simulation time. We have implemented a pipelining mechanism for data transmission to address this issue, as depicted in Figure 6. It is important to note that there are no dependencies for write operations between the calculation at time t and the data from time t − 1. Essentially, the calculation at time t only requires retrieving data from time t − 1 without modifying its values. As a result, the GPU can concurrently perform calculations for the current time step while transmitting results from the previous time step at each time step.

To implement this part, we used GPU streams provided by CUDA in our programming. Specifically, we employed two streams on the GPU: one responsible for the simulation calculations and the other for data transfer. It is important to note that since the computation may overwrite variables, using the same variable for both calculation and transfer could lead to data corruption. To address this issue, we created a duplicate of each variable that needs to be transferred to ensure data synchronization. At the same time, to minimize unnecessary data synchronization, the variable and its duplicate are alternately used at each time step. Specifically, if the original variable stores the result of the current time step at time t, its duplicate will store the result from time t − 1. Then, at time t + 1, the duplicate will store the result for the t + 1 time step while the original variable is used for data transfer. This functionality can be implemented using a bit-wise AND operation. For each variable that needs to be transferred, we use t&1 as the index to select between the variable and its duplicate. Since the time step t increases incrementally, the index will alternately cycle between 0 and 1. Therefore, for stream0 (the stream used for computation), we use t&1 as the index for variable calculations, and for stream1 (the stream used for data transfer), we use (t+1)&1 as the index for variable transfer. In this way, the index for stream0 alternates between 0 and 1, while for stream1, the index alternates between 1 and 0. By employing this method, we avoid data synchronization, thereby reducing the simulation runtime.

## 3. Experiment Results

The experimental procedures in this study were conducted on a server system equipped with an Intel i7-7700 CPU and Nvidia GeForce GTX 1080 GPU. Comprehensive information about the hardware specifications is detailed in Table 2, while Table 3 describes the software configuration. The GPU parameters related to some of the runtime configuration settings are shown in Table 4. Given that CUDA supports the C++ programming language, the simulation implementation in this study primarily leverages C++ programming paradigms.

### 3.1. The Validation Experiment of GPU Parallelization

**Figure 7:**
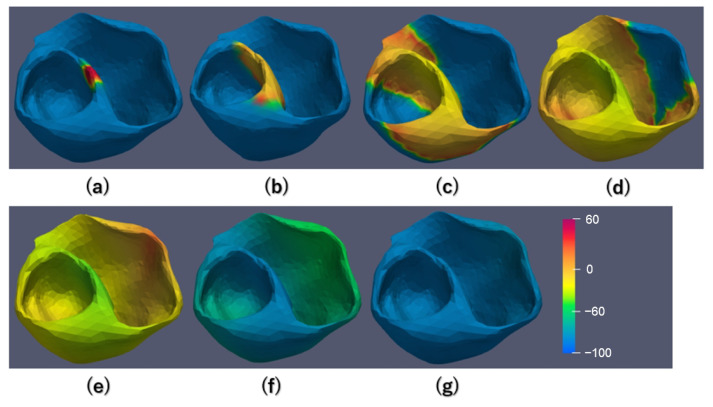
Snapshots of propagating action potentials across the whole 3D mesh. (**a**): 2 ms; (**b**): 10 ms; (**c**): 20 ms; (**d**): 30 ms; (**e**): 40 ms; (**f**): 90 ms; (**g**): 100 ms.

In order to ensure the accuracy of our implementation, we conducted a brief simulation on a small 3D mesh. During this simulation, the total simulation time was set to 150 ms, with a time step of 0.005 ms. The validation results can be found in Figure 7. This validation was carried out on a 3D mesh consisting of 4168 nodes, with solid stimulation applied to the upper central region of the mesh. The potentials of all nodes were recorded at specific time points: 2 ms, 10 ms, 20 ms, 30 ms, 40 ms, 90 ms, and 100 ms. The recorded data were then visualized using heat maps.

The validation experiment results are as follows: Initially, action potentials were observed in the upper central region in response to intense stimulation, as shown in Figure 7a. As the diffusion process progressed, the electrical signal spread to adjacent nodes, causing them to exhibit action potentials. The sequential spread of action potentials across the grid is shown in Figure 7b–e. By 40 ms, all nodes in the entire heart model were activated, indicating successful signal propagation. Subsequently, all nodes returned to a resting state. These experimental results align with the sequential program results, confirming our parallel implementation’s accuracy.

### 3.2. The Acceleration Performance of GPU Parallelization

**Figure 8:**
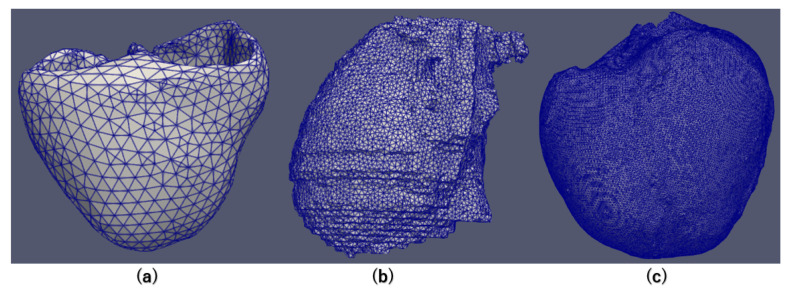
The structure of small/mid/large mesh. (**a**): small mesh; (**b**): mid mesh; (**c**): large mesh.

To illustrate the performance differences between CPU and GPU, we conducted extensive simulations using various 3D meshes, as detailed in Figure 8. This study encompassed three distinct categories of 3D grids, each precisely characterized by its number of nodes. Table 5 concisely presents comprehensive information about each grid. These grids are carefully classified into small, medium, and large categories based on node counts. Specifically, small grids consist of 4168 nodes, while large grids encompass 646,627 nodes. These diverse grids were employed to carry out simulations at various scales. The time step may vary depending on the cell model and precision. Our electrocardiogram research focuses more on changes in the action potential morphology, such as early after-depolarization (EAD), delayed after-depolarization (DAD), and re-entry phenomena. Therefore, the precision requirement is not very high, and we selected a time step of 0.005 ms for our experiments. With this time step, the simulation can be performed efficiently without encountering issues like overflow or NaN errors related to precision.

The relevant simulation parameters are summarized in Table 6. We chose the CPU serial program with the improved mesh storage as the baseline for our comparative experiments, as this approach applies to both CPU and GPU simulation programs. The implementation details have been discussed in the preceding sections.

**Figure 9:**
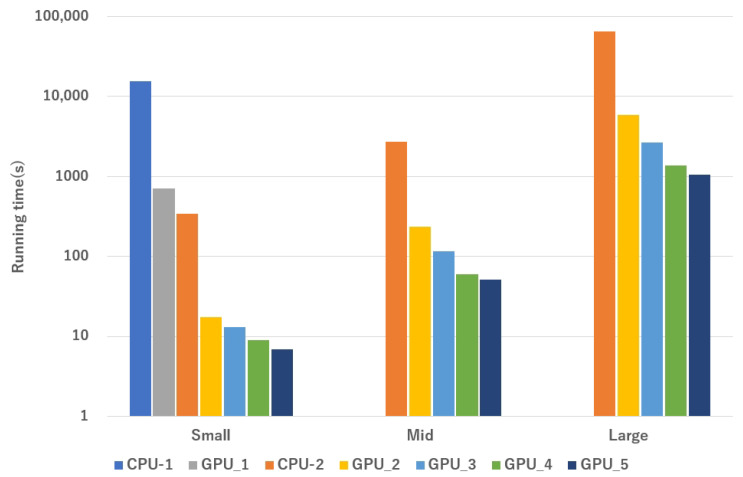
Running time of each algorithm for different scale cardiac simulation. CPU_1: Serial program on CPU; CPU_2: CPU_1 + adjacency list; GPU_1: GPU parallelization without any optimization strategies; GPU_2: GPU parallelization with adjacency list + node index regeneration; GPU_3: GPU_2 + improved data storage; GPU_4: GPU_3 + improved algorithm; GPU_5: GPU_4 + async data transfer.

The simulation runtimes for three different mesh scales, using various optimization strategies on both CPU and GPU platforms, are shown in Figure 9 and Table 7. Due to the long runtimes, only performance data for the small mesh are presented, excluding the adjacency list optimization. The results demonstrate the significant advantage of GPUs over CPUs in cardiac simulations, mainly due to the GPU’s SIMD capabilities, which greatly accelerate the simulation process.

Furthermore, choosing the correct storage method for the mesh is important because it directly affects how quickly the neighboring nodes can be retrieved. Optimizing cache hit rates and streamlining data access patterns, whether focused on concentration or intensity, significantly impact simulation runtimes. When comparing the runtimes of Algo GPU_2 and Algo GPU_3 at different mesh scales, it becomes clear that flattening the data structure leads to substantial improvements in runtime efficiency as the simulation scale increases.

**Figure 10:**
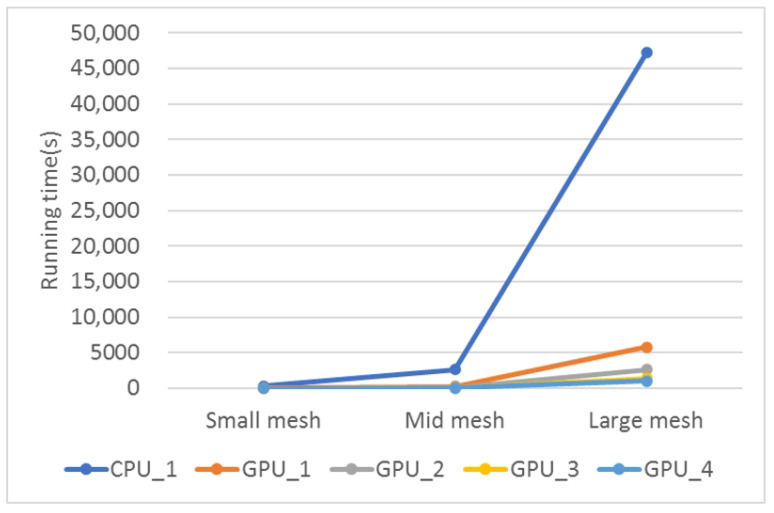
The increasing trend of running time for different meshes. GPU_1: GPU parallelization with adjacency list + node index regeneration; GPU_2: GPU_1 + improved data storage; GPU_3: GPU_2 + improved algorithm; GPU_4: GPU_3 + async data transfer.

Figure 10 illustrates the increasing trend in runtime as the simulation scale expands, comparing the performance of CPU and GPU simulation programs. The figure clearly shows the exponential growth in runtime for the CPU sequential program. The ODE computation is almost proportional to the simulation scale for the CPU serial program. During the propagation phase, the average computation time per node also increases due to the growing number of neighboring nodes. As a result, for simulations involving a large mesh, the CPU program requires an impractical duration of approximately 50,000 s to complete, rendering it inefficient.

The GPU programs consistently show significant simulation efficiency compared with their CPU counterparts. Several optimizations, including improvements in storage approach, algorithm refinement, and asynchronous transmission, contribute significantly to this efficiency. As a result, a large mesh simulation concludes in approximately 1000 s, highlighting the substantial advantage of GPU-based simulations.

Due to the influence of hardware and software environments, it is not reasonable to directly compare the parallel results with those of other studies. Additionally, due to differences in the original algorithms, it is challenging to directly apply others’ parallel methods to our algorithm. Conversely, some of the optimization strategies presented in this paper may yield minimal benefits when applied to others’ algorithms. Therefore, reproducing others’ work is quite difficult, and comparing our parallel results with those of other studies is also very challenging. Based on the reasons mentioned above, we computed the acceleration ratio using the subsequent formula to further emphasize the exceptional efficiency of GPUs:

(7) $$s=\frac{t_{\mathrm{serial}\mathrm{program}}}{t_{\mathrm{parallelized}\mathrm{program}}}$$

**Figure 11:**
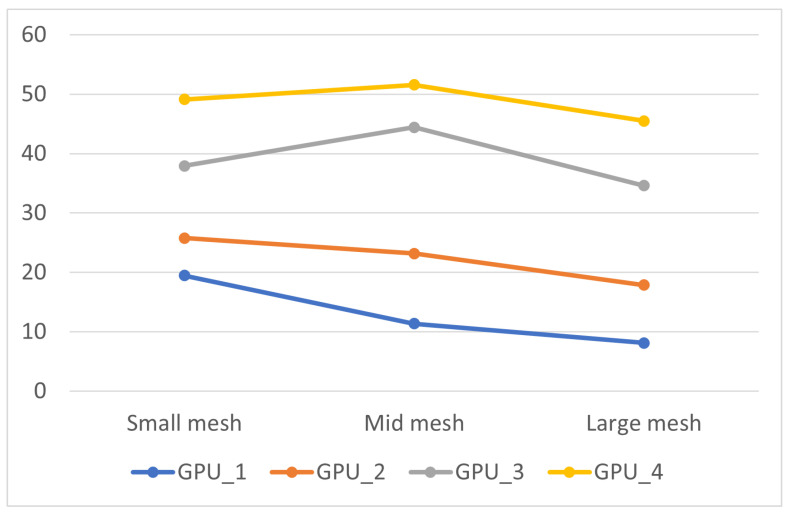
The speedup ratio of the algorithm. GPU_1: GPU parallelization with adjacency list + node index regeneration; GPU_2: GPU_1 + improved data storage; GPU_3: GPU_2 + improved algorithm; GPU_4: GPU_3 + async data transfer.

The results shown in Figure 11 support the claim that the GPU parallel program, even without any optimization, achieves about ten times faster than the CPU. With storage optimizations, this speedup increases to around 20-fold. By implementing an improved algorithm that allows simultaneous execution of ODE and PDE computations, the speedup reaches approximately 40-fold. Finally, integrating pipelined asynchronous data transmission stabilizes the speedup at around 55-fold.

However, the chart shows that as the computational scale increases, the speedup diminishes to some extent as the data size grows, despite various optimization strategies. This happens because most optimization strategies focus on reducing the number of global memory accesses through efficient caching in the ODE calculation portion with a smaller impact on the PDE component. Moreover, as the number of cell nodes increases, it becomes challenging to ensure that each thread is responsible for the calculations of only one node. In our GPU setup, when simulating a large mesh, each thread handles approximately five nodes, while in a small mesh, each thread handles only one node. This difference leads to decreased performance compared with the condition where each thread manages a single node.

Another reason for this performance variation is the different strategies employed in CPU and GPU simulations. We reduce some ODE calculations in CPU simulations by checking if cell nodes are at rest. However, our tests showed this approach is less effective on the GPU. The GPU’s inherent Single Instruction, Multiple Data (SIMD) execution mode requires threads within the same warp to execute the same instruction simultaneously. Excessive branching disrupts this feature, as some threads within the same warp may follow different branches, leading to suboptimal parallelism. As a result, while the strategy effectively avoids some ODE calculations for certain nodes on the CPU, it leaves nodes idle while others are being computed on the GPU. Therefore, we did not use this strategy on the GPU, resulting in more ODE calculations per node in the GPU simulation. Although this difference decreases as the current diffuses, it still impacts the results, especially as the simulation scale increases.

Also, as the computational scale increases, the time required to transfer results back to the CPU at each time step increases. Although we aim to mitigate this time overhead through a pipelining approach, this technique has limitations as the computational load grows.

In order to accurately demonstrate the computational acceleration of the GPU, a simple comparison of speedup ratios is not sufficient. To showcase the GPU’s computational performance, we calculated the average time spent per node, as displayed in Table 8. In this calculation, we excluded the time spent on data transmission and focused only on the running time of the computational part.

Based on the data in the table, it’s clear that when it comes to GPU parallelization, the GPU_1 program, which utilizes adjacency lists, shows a much higher average computation time per node at large scales than at small scales. This difference is mainly due to frequent access to global memory. However, after optimizing data storage in GPU_2, the performance at large scales becomes roughly comparable to that at mid-scales. While there is still a performance difference compared with small scales, it has been significantly reduced.

After implementing the enhanced algorithm, the average computation time per node remains between 0.66 and 0.69 ms. This effectively resolves the problem of decreased cache hit rate resulting from the expansion of the simulation scale. The GPU’s computational performance remains consistent across all three scales. These findings demonstrate the effectiveness of the optimization strategies in improving computational performance.

**Figure 12:**
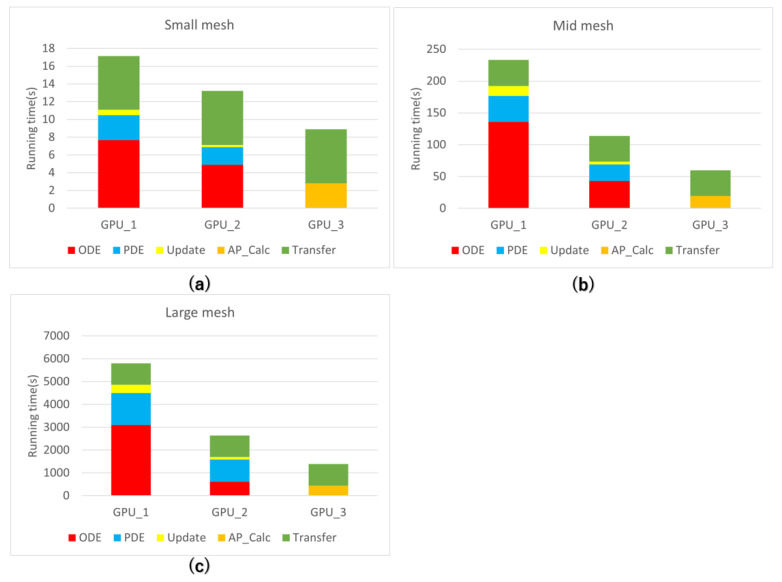
Time consumption for each part of the algorithm. GPU_1: GPU parallelization with adjacency list + node index regeneration; GPU_2: GPU_1 + improved data storage; GPU_3: GPU_2 + improved algorithm. (**a**): Time consumption on the small mesh. (**b**): Time consumption on the mid mesh. (**c**): Time consumption on the large mesh.

In order to show the impact of the improved data storage method and algorithm on parallel efficiency, we analyzed the time allocation for each part of the simulation. We then represented the results in Figure 12. Looking at Figure 12a–c, it’s clear that in the traditional numerical solution approach, the ODE component takes up a significant amount of the total simulation time, especially in programs with unoptimized data storage. However, with the implementation of the improved storage strategy, the ODE component’s computational time is notably reduced. Specifically, for simulations involving a large mesh, the ODE computation time, initially around 3000 s, has been reduced to just over 600 s. On the other hand, the improvement in data storage has a relatively small effect on the computation time of the PDE component. This difference primarily comes from the GPU’s frequent access to various parameters within the LR model during ODE computations, compared with the reliance of PDE computations solely on the model’s action potential.

The optimized algorithm combines the calculations of ODE and PDE into a single function, eliminating the need for synchronization and update processes for the entire grid. This results in a significant reduction in simulation runtime. The original 5000-s simulation computation period has been reduced to approximately 450 s after implementing optimizations in both the algorithm and storage strategy.

**Figure 13:**
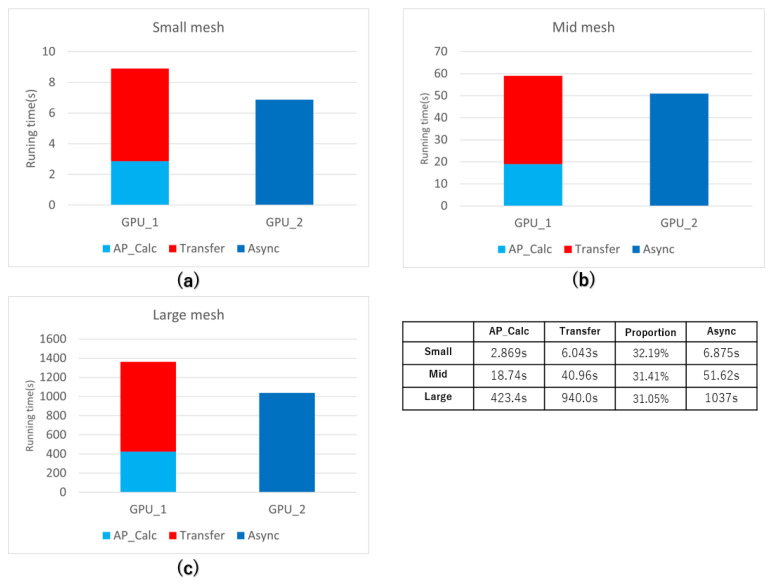
Detailed comparison of the time consumption of sync and async data transfer. (**a**): Time consumption on the small mesh. (**b**): Time consumption on the mid mesh. (**c**): Time consumption on the large mesh.

The comparison in Figure 13a–c shows the time consumption of synchronous and asynchronous data transfer at different simulation scales. The corresponding tables demonstrate that, after several systematic optimizations, the program without pipeline asynchronous transfers spends only 30% of the total simulation duration on the computation phase. Approximately 70% of the time is allocated to data transfer activities. The pipeline implementation involves concurrent interleaving of result transfers from the previous time step with the ongoing computation process, which significantly enhances program execution efficiency. In extensive simulations, asynchronous transfers combined with computations are only marginally slower, approximately 10%, compared with direct data transfers, highlighting the importance of pipeline transfers.

### 3.3. Effects of Optimization Strategies on Cache Hit Rate

The cache hit rate is an important measure of how efficiently a program accesses global memory during simulations. A higher cache hit rate means that most of the data needed for calculations is already stored in the cache, reducing the need to frequently fetch data from global memory. Since accessing global memory is significantly slower than accessing cache memory, a higher cache hit rate means less time spent repeatedly reading data, leading to overall improved runtime efficiency of the program.

Table 9 provides a comprehensive overview of the L2 cache hit rates for the GPU parallel simulation program following data flattening and algorithmic enhancements. The results show that all three strategies have high cache hit rates for small simulation scales. However, as the simulation scale increases, the program without data flattening has a cache hit rate of less than 20% during the ODE component’s computation. On the other hand, using an improved data storage method leads to a noticeable increase in cache hit rates. This improvement is further enhanced by integrating an improved algorithm that combines the computation of the ODE and PDE components, resulting in a subsequent enhancement of the cache hit rates.

The increased hit rates lead to fewer global memory read operations, resulting in faster simulation speed. These results highlight the significance of efficient data storage and algorithmic optimizations in improving cache hit rates and enhancing the performance of GPU parallel simulation programs.

### 3.4. Effects of Data Storage on GPU Memory Usage

The Luo–Rudy model has about 60 parameters, and storing the model for one node usually takes around 480 bytes. This is calculated from 60 parameters, each using 8 bytes. After analyzing the model, it was found that approximately 20 parameters have a constant value throughout the simulation. These constant parameters can be efficiently stored in the GPU’s texture memory, requiring only one storage instance. Using this method, the number of model parameters per node has been reduced from 60 to 40, resulting in a memory allocation reduction from 480 bytes to 320 bytes. This optimization represents a 33% improvement in memory usage.

For a large mesh consisting of 646,627 nodes, the conventional storage approach would need around 300 MB of memory. However, the optimized strategy requires only 200 MB of memory space. This optimization is especially beneficial for complex models with many internal parameters. Constant extraction methods are an effective way to significantly decrease memory usage.

### 3.5. Error Analysis of Improved Algorithm

**Figure 14:**
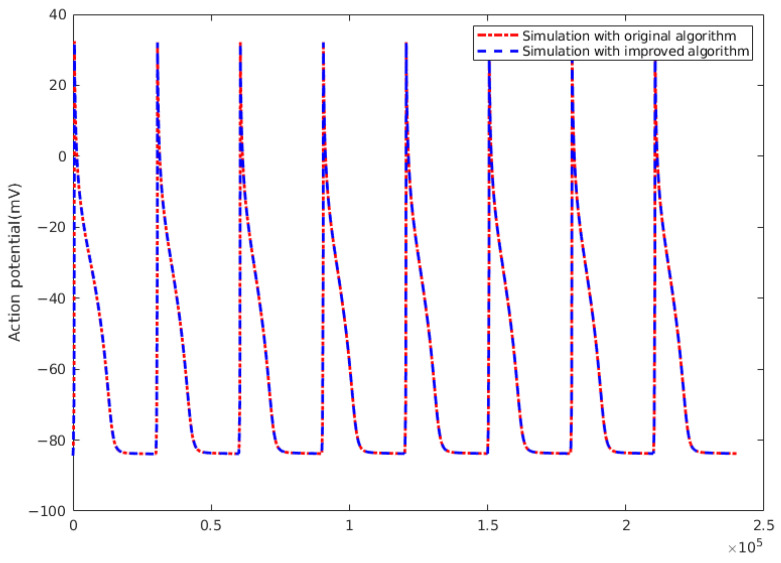
Action potential of one node between numerical method and improved method.

The image in Figure 14 and the data in Table 10 show the differences between the original numerical solution method and the enhanced numerical solution approach. As shown in the figure, there are very small visual differences between the two algorithms. To conduct a more comprehensive comparison between the two methods, we have used three different evaluation metrics: Mean Squared Error (MSE), Root Mean Squared Error (RMSE), and Mean Absolute Error (MAE). The calculation methods for these metrics are explained below:

- Mean Squared Error (MSE): $\frac{1}{n}\sum_{i=1}^{n}{\left(y_{i}-\hat{y_{i}}\right)}^{2}$
- Root Mean Squared Error (RMSE): $\sqrt{\frac{1}{n}\sum_{i=1}^{n}{\left(y_{i}-\hat{y_{i}}\right)}^{2}}$
- Mean Absolute Error (MAE): $\frac{1}{n}\sum_{i=1}^{n}\left|\left(y_{i}-\hat{y_{i}}\right)\right|$

The table shows metrics for time steps of 0.005 ms and 0.001 ms. As the time step decreases, the differences resulting from the algorithmic modification also decrease. The Mean Absolute Error (MAE) values at 0.005 ms and 0.001 ms are 0.0872 and 0.0171, respectively. Compared with the amplitude of action potential values ranging from −100 to 40, these MAE values are very small. To better understand how error changes with simulation time, we conducted simulations of different simulation times and calculated the error, with the results shown in Table 11. The results show that the error changes very slowly as simulation time increases and remains within an acceptable range. The main reason for this phenomenon is that after generating an action potential, the cell enters a refractory period during which it is at a resting potential and does not generate new action potentials in response to stimuli, resulting in minimal changes in potential. Based on these experimental results, we believe that the error can still be well controlled even for longer simulations. Additionally, the algorithm change has led to a roughly 50% reduction in simulation runtime. This demonstrates the strategy’s effectiveness, which involves delaying the influence of the PDE. This approach maintains precision and is highly reliable and efficient.

### 3.6. The Scalability of GPU Parallel Approach

To further demonstrate the scalability of the parallel method in this paper, we conducted simulations using different cell models.

Currently, there are two main types of cell models. One type is based on modeling the action potential waveform. These models often have simple expressions with parameters that lack physiological significance, focusing on simulating the cell’s action potential while lacking a more profound representation of intracellular electrophysiological activity. The other type is derived from the Hodgkin and Huxley theory. These models express electrophysiological activity through the modeling of ion channels. Compared with the former, this type of cell model is more suitable for exploring the internal mechanisms of cells and can also simulate different cell pathologies or mutations by adjusting the relevant parameters of the ion channels.

When selecting cell models, we mainly chose those based on the Hodgkin and Huxley theory. The reason is twofold: The first type of cell model has relatively simple expressions and does not require much computational resources, so simulations can be efficiently completed even on CPUs. On the other hand, the application of this type of model is quite limited, as it only models the action potential waveform and cannot be used to explore the internal mechanisms of cardiac electrophysiology. Through comparison, we ultimately selected the Luo–Rudy 1991 model [28], the Stewart Zhang 2009 PF model [31], and the Takeuchi HL1 model [32], three different cell models, to demonstrate the scalability of the proposed method concerning cell models. All three cell models are derived from the Hodgkin and Huxley theory. The difference is that each cell model has a different number of ion channels and the expressions for each ion channel vary.

The runtime is listed in Table 12. As can be seen, despite using different cell models, GPU parallelization still achieved excellent acceleration. When using the Takeuchi HL1 model, the CPU serial program took almost 46 h to complete the simulation, which is highly unacceptable. In contrast, GPU parallelization reduced the time to only about 40 min. Furthermore, we calculated the cache hit rate for different cell models. The experimental results listed in Table 13 indicate that as the complexity of the model increases and more ion channels are introduced, the cache hit rate gradually decreases when the memory optimization strategy is not applied. This decline was caused by increased parameters, leading to more fragmented memory storage. However, when the memory optimization strategy was employed, the cache hit rate remained relatively high. The reason is that, despite the increase in the number of parameters, the same type of parameters were still stored continuously in memory. Therefore, the memory optimization strategy remained effective across different cell models and varying numbers of parameters.

To demonstrate the performance of our method on large-scale models, we used the “Oxford Rabbit Heart” [33], a highly detailed MR-based rabbit mesh with 4 million elements, making it one of the highest-resolution cardiac meshes in the world, for simulation. Additionally, we performed simulations on a coarse-resolution version with 40,000 nodes from the “Oxford Rabbit Heart” mesh. We also used Chaste [34], an open-source simulation library that supports CPU parallelization, to simulate both meshes. By comparing the runtime of our method with Chaste, we demonstrate the efficiency of GPUs and the scalability of our method on large-scale models.

In the Chaste simulation, we used eight cores for CPU parallelization. The runtime for the two meshes is shown in Table 14. As the table shows, even with CPU parallelization, a significant amount of time is still required to complete the simulation for large-scale meshes. For the 4-million-node mesh, Chaste took approximately 31 h to simulate 100 ms, which is unacceptable for scenarios requiring frequent parameter adjustments. In contrast, our method only took about 3 h to complete a short-term simulation, which is much more acceptable. Moreover, the simulation on the large-scale mesh further demonstrated the scalability of our method.

## 4. Discussion

This study focuses on parallelizing three-dimensional cardiac electrophysiology simulations using a single GPU. After establishing the computational framework, optimization procedures were systematically applied, focusing on the mesh’s unique structural attributes and leveraging the GPU’s inherent capabilities. These optimization strategies were applied to the preprocessing, simulation, and post-processing phases, leading to a significant improvement in the efficiency of the simulations compared with unoptimized GPU parallel programs. Consequently, this enhancement has allowed extensive cardiac electrophysiology simulations on a single graphics card.

The experiments provide empirical evidence that the most significant improvement in operational efficiency among this series of optimizations comes from consolidating adjacency lists. The application of adjacency lists directly reduces the time complexity for accessing neighboring nodes, transitioning from O(n) to O(m), where n represents the number of tetrahedral elements and m represents the number of neighboring nodes. Simultaneously, node reordering, enhanced storage formats, and algorithm improvements were designed to increase cache hit rates and reduce access to global memory.

Furthermore, optimized storage formats help alleviate memory overhead. Empirical results highlight that when using the LR model, enhanced storage formats can reduce memory consumption by approximately 30%. The strategy of pipeline data transfer aims to mitigate the time overhead caused by data transfer through concurrent computations. Experimental results show that after optimization, the computation phase accounts for only 30% of the entire simulation runtime.

Furthermore, the integration of the pipeline does not significantly improve simulation efficiency. However, with further research into model complexity and the combination of additional ionic currents, the computational complexity of the simulation is expected to increase. In such cases, the advantages of the pipeline are expected to gradually become more evident.

The experiments provide empirical evidence that there is a significant improvement in operational efficiency when using GPU programs compared with CPU programs. The experimental results show that, with the same adjacency list, the GPU program is approximately 10 to 20 times faster than the CPU program. After a series of optimizations, the speedup increases to about 50 times. Large-scale cardiac electrophysiology simulations that took 14 h can now be accomplished on the GPU in approximately 15 min. These experimental findings highlight GPUs’ substantial impact on cardiac electrophysiology simulation research.

Compared with other studies, this research focuses on parallelizing cardiac electrophysiology simulations on a single GPU. The study comprehensively analyzes a series of optimizations for the three-dimensional simulation implementation, from preprocessing to post-processing. In particular, the paper emphasizes using an improved adjacency list for mesh storage to efficiently access neighboring nodes efficiently. Additionally, it employs static arrays instead of traditional adjacency lists to ensure cache hit rates. The paper also introduces an improvement that utilizes data from the previous time step for PDE updates, decoupling the calculations of ODE and PDE, thus reducing the time required for synchronization between threads. Unlike other parallel methods, this paper also addresses data transmission after computation, reducing the time overhead in this part through a pipelining approach.

Additionally, the parallel method proposed in this paper is also applicable to other cell models and approaches. The cell model used in this study is the Luo–Rudy model, which is based on the Hodgkin and Huxley theory. In this type of model, the total current is expressed as the sum of the currents generated by various ion channels, with each ion channel represented by multiple ODEs. Subsequent models of this kind have introduced new ion channels or improved the expressions of existing ones without altering the overall structure.

These models often involve a large number of parameters, and during ODE calculations, each ion channel’s parameters must be updated. Therefore, reducing memory access is key to speeding up the simulation of these models. The memory optimization strategy used in this paper thus provides excellent acceleration performance for all cell models based on the Hodgkin and Huxley theory.

The mesh optimization strategy, aimed at quickly locating neighboring nodes, is independent of the specific implementation of potential propagation, making it applicable to other propagation algorithms as well. Finally, experimental results have shown that the algorithmic improvements provide efficient acceleration for the computational model used in this paper while maintaining a certain level of accuracy. If other propagation algorithms are employed, additional improvements and optimizations tailored to the specific algorithm would be necessary.

The acceleration achieved by running cardiac simulations on a single GPU may not be as impressive as using multiple GPUs. However, it is still highly cost-effective and efficient for small to medium-scale simulations. This paper also provides discussion on the scalability of the parallel method; our simulation results on the large mesh also demonstrate that this approach can still achieve significant acceleration in larger simulations. For larger-scale simulations involving millions of nodes, the computational load for the ODE part does not increase, as the ODEs reflect the inherent electrophysiological mechanisms within the cell. In the propagation phase, the potential at each node is influenced only by its neighboring nodes, and the number of these neighboring nodes increases only slightly. Therefore, the average computational load per node will not increase significantly in larger-scale simulations. Nevertheless, the number of cores on a GPU is quite limited. The latest Nvidia 4090 Ti, for example, has 18,176 CUDA cores, which is far fewer than the number of nodes in the simulation. As a result, each CUDA core must handle computations for dozens of nodes. When dealing with millions of nodes, a single CUDA core may even need to compute for hundreds of nodes. Therefore, as the simulation scale reaches a certain point, the number of nodes each CUDA core has to process can be considered proportional to the total number of nodes. In this condition, the parallel acceleration effect becomes constrained. Despite the aforementioned limitations of a single GPU, the computational power of a single GPU is adequate to effectively support the majority of current cardiac electrophysiology simulations, providing significant advantages in terms of cost and maintenance compared with multiple GPUs or GPU clusters. However, using a GPU cluster for acceleration remains a viable choice for simulations requiring a large amount of memory. Nevertheless, GPUs remain the preferred platform for conducting cardiac electrophysiology simulations, highlighting their significant contributions.

## 5. Conclusions

In this research, we proposed an approach focused on speeding up 3D cardiac simulations using GPU computing. Our investigations provide strong evidence that demonstrates the significant advantages of GPU computing over traditional CPU-based methods. The noticeable increase in speed and reduction in overall simulation runtimes indicate this comparative advantage. Furthermore, our study proves that GPUs maintain remarkable performance efficiency even when conducting large-scale cardiac electrophysiology simulations. This emphasizes the potential of GPUs as a powerful platform for advancing cardiac simulation work, showcasing their ability to significantly improve computational efficiency and accelerate scientific progress in this field.

## Figures and Tables

**Table 1:** Running time and transfer time for different meshes on GPU platform (only adjacency list used).

Node Number	Tetrahedron Number	Time Step	Print Step	Running Time	Transfer Time	Proportion
4168	17,937	0.005 ms	0.005 ms	17.328 ms	6.034 ms	34.82%
28,187	112,790	0.005 ms	0.005 ms	234.402 ms	40.974 ms	17.48%
646,627	3,475,122	0.005 ms	0.005 ms	5807.041 ms	940.01 ms	16.19%

**Table 2:** Hardware configuration of experiment environment.

Hardware	Description
CPU	Intel i7-7700 3.60 GHz * 8
GPU	Nvidia GeForce GTX 1080 SSE2
Memory	32 GB DDR4
Disk	520 GB SSD

**Table 3:** Software configuration of experiment environment.

Software	Description
Operating System	Ubuntu 18.04.5 LTS
CUDA	CUDA v10.0.130
g++	g++ 7.5.0

**Table 4:** Parameters of GPU.

Name	Value
GPU Memory	8106 MBytes
Cores	2560 cuda cores
Warp Size	32
Max threads/Multiprocessor	2048
Max threads/Block	1024
L2 cache size	2 MBytes

**Table 5:** Three-dimensional mesh description.

Mesh	Nodes	Tetrahedrons
Small	4168	17,937
Mid	28,187	112,790
Large	646,627	3,475,122

**Table 6:** Simulation configuration.

Name	Value
Numerical method	Runge–Kutta method
Simulation time	100 ms
Time step	0.005 ms
Threads/Block	512
Block/Grid	256

**Table 7:** Running time of each algorithm for different scale cardiac simulation. CPU_1: Serial program on CPU; CPU_2: CPU_1 + adjacency list; GPU_1: GPU parallelization without any optimization strategies; GPU_2: GPU parallelization with adjacency list + node index regeneration; GPU_3: GPU_2 + improved data storage; GPU_4: GPU_3 + improved algorithm; GPU_5: GPU_4 + async data transfer.

Algo	Small Mesh	Mid Mesh	Large Mesh
CPU_1	15,333.7 s		
CPU_2	337.573 s	2665.14 s	47,261.34 s
GPU_1	700.573 s		
GPU_2	17.328 s	234.402 s	5807.041 s
GPU_3	13.092 s	114.881 s	2638.647 s
GPU_4	8.903 s	59.974 s	1363.397 s
GPU_5	6.875 s	51.672 s	1037.327 s

**Table 8:** The average computation time per node of each algorithm for different scale cardiac simulation. CPU: serial program on CPU without ODE detection strategy; GPU_1: GPU parallelization with adjacency list + node index regeneration; GPU_2: GPU_1 + improved data storage; GPU_3: GPU_2 + improved algorithm.

Algo	Small Mesh	Mid Mesh	Large Mesh
CPU	81.02 ms	94.83 ms	98.89 ms
GPU_1	2.66 ms	6.81 ms	7.51 ms
GPU_2	1.70 ms	2.61 ms	2.62 ms
GPU_3	0.66 ms	0.69 ms	0.69 ms

**Table 9:** L2 cache hit rates for each part of the algorithm. GPU_1: GPU parallelization with adjacency list + node index regeneration; GPU_2: GPU_1 + improved data storage; GPU_3: GPU_2 + improved algorithm.

Algo	GPU_1			GPU_2			GPU_3
	ODE	PDE	Update	ODE	PDE	Update	AP_Calc
Small	71.38%	77.74%	51.94%	80.75%	78.85%	72.40%	88.17%
Mid	15.03%	35.98%	0.08%	46.52%	36.06%	1.15%	74.75%
Large	17.71%	18.72%	0.15%	57.21%	22.74%	0.36%	64.22%

**Table 10:** Error metrics of improved algorithm.

Time Step	MSE	RMSE	MAE
0.005 ms	0.0173	0.1314	0.0872
0.001 ms	0.000665	0.0258	0.0171

**Table 11:** Error metrics of improved algorithm under different simulation times. Time step: 0.005 ms.

Simulation Time	MSE	RMSE	MAE
500 ms	0.0147	0.1212	0.0867
1 s	0.0153	0.1237	0.0871
5 s	0.0144	0.1183	0.0860
10 s	0.0217	0.1473	0.0893
60 s	0.0194	0.1393	0.0889

**Table 12:** Running time of different cell models on large mesh. A: Luo–Rudy 1991 model; B: Stewart Zhang 2009 PF model; C: Takeuchi HL1 model; CPU: serial program on CPU; GPU: parallel program with all optimization.

Model	Ion Channel	Parameters	CPU	GPU
A	6	60	47,383.51 s	1041.57 s
B	14	130	109,213.83 s	1871.69 s
C	17	190	164,713.91 s	2364.14 s

**Table 13:** L2 cache hit rates for each part of the algorithm. A: Luo–Rudy 1991 model; B: Stewart Zhang 2009 PF model; C: Takeuchi HL1 model; GPU_1: GPU parallelization with adjacency list + node index regeneration; GPU_2: GPU_1 + improved data storage; GPU_3: GPU_2 + improved algorithm.

Model	GPU_1			GPU_2			GPU_3
	ODE	PDE	Update	ODE	PDE	Update	AP_Calc
A	16.93%	18.46%	0.15%	58.75%	24.69%	0.37%	65.35%
B	12.44%	17.73%	0.13%	54.13%	23.91%	0.35%	63.74%
C	10.12%	15.59%	0.09%	51.35%	23.06%	0.33%	62.62%

**Table 14:** Running time of different meshes. Cell model: Luo–Rudy 1991 model; simulation time: 100 ms; timestep: 0.01 ms.

Mesh	Nodes	Chaste-8cores	GPU
Oxford Rabbit Heart (Coarse Version)	431,990	2.4 h	863.27 s
Oxford Rabbit Heart (Fine Version)	4,283,195	31 h	10,124.96 s

## Data Availability

Data are contained within the article.
